# Exploring the Theological Context to Domestic and Family Violence

**DOI:** 10.1177/10778012241254849

**Published:** 2024-05-24

**Authors:** Josephine Clarke, Sarah Wendt, Wendy Mayer

**Affiliations:** 1 Department of Social Work, School of Health Sciences, 2281 University of Melbourne, Melbourne, Victoria, Australia; 2 1065 College of Education, Psychology and Social Work, Flinders University, Adelaide, Australia; 3 95552 University of Divinity, Melbourne, Victoria, Australia

**Keywords:** religion, gender, domestic violence

## Abstract

This article explores the theological drivers of domestic and family violence (DFV)—specifically intimate partner violence—by engaging with ecclesiastical beliefs and practices of the Lutheran Church of Australia (LCA). Key theological, policy, and public documents were analyzed to understand gender relations, gender roles, marriage, ordination, ethical behavior, and responses to DFV. Findings from the document analysis demonstrate church practice and policy reforms underway in addressing violence against women and supporting safety. Simultaneously, the documents show contested and troubled positions regarding gender relations, the theological context to gender roles and responsibilities and the church's journey of debating (re)configuration of its organizational structure and responsibilities.

## Introduction

Domestic and family violence (DFV)^1^—specifically intimate partner violence—is a profound violation of victim/survivor health, well-being, safety, security, and citizenship (Franzway et al., 2019; Wendt & Zannettino, 2015). Data indicate the gendered nature of DFV. Drawing on Australian statistics, the Australian National Plan to End Violence against Women and Children 2022–2032 describes how “most victims and survivors are women … One in 4 women has experienced intimate partner violence since the age of 15” (Commonwealth of Australia, 2022, p. 39).

Increasingly religious organizations and communities^2^ are recognizing they have a role in identifying, preventing, and responding to DFV (Anglican Church of Australia, 2021; Davis et al., 2021; in Touch, 2020; LCA, 2020). This includes churches and faith-based organizations developing various strategies and campaigns to recognize and address intimate partner violence. Initiatives include activities to raise awareness of what is DFV; training clergy, lay workers, and key personnel; developing inter-faith strategic connections; developing organizational policies and procedures that clarify church-based and institutional legal responsibilities regarding responses and provision of support to those experiencing and impacted by violence and abuse, and to promote safety.^3^ Further, some religious organizations have expressed their concern that there are connections to be made between specific ecclesiastical beliefs and practices that have detrimentally contributed to DFV and specifically, male perpetrator violence against women (see Anglican Church of Australia, 2021). Some religious organizations have also undertaken or commissioned research to further understand gender-based violence in their communities (Powell & Pepper, 2021). At the same time, there is evidence of faith leader and community reluctance to recognize and discuss issues relating to DFV (Knickmeyer et al., 2010; Truong et al., 2020).

In Australia in the 2021 census, 43.9% of the population identified with Christianity (the dominant albeit declining religious affiliation) (Australian Bureau of Statistics, 2022a). The Lutheran Church of Australia (LCA) is a Christian denomination^4^ that represents 1.3% of the total Australian Christian population (Australian Bureau of Statistics, 2002b). The LCA has engaged in processes and practices to recognize, prevent, and address the issue of DFV. This article reports on research findings from an analysis of documents produced by the LCA that articulate the LCA's understanding of the problem of DFV and its role and responsibility in preventing and responding to this type of violence. This document analysis method includes attention to the LCA's expression of gender relations, gender roles, marriage, ordination, and ethical behavior, with particular attention to the LCA's use of scripture and theological framing of the problem of DFV.

The document analysis was conducted as part of a larger research project exploring religion and men's perpetration of DFV (Wendt et al., 2023a).^5^ The research proposal was established in response to the LCA approaching the research project's Chief Investigator about the opportunity to conduct research that will support the work of the LCA's domestic violence (DV) Taskforce and the capacity of the LCA to address DFV. The research focus on male perpetration was consolidated through conversation with the LCA's DV Taskforce and members provided support and advice throughout the life of the research project. The LCA DV Taskforce and research project team members met regularly; this provided researchers with the opportunity to provide updates, discuss preliminary findings, and receive feedback and questions from Taskforce members.

The research aims included: describing how Christian beliefs and practices can contribute to and perpetuate DFV; determine how men understand their use of violence in intimate and family relationships within the context of their Christian (Lutheran) beliefs and practices; explore men's help-seeking experiences inside their church settings; and further, identify strategies to enable church settings to engage with men to enable attitudinal and behavior change to stop using violence. This article reports on findings from analysis of documents conducted to understand how the LCA has discussed and reviewed its role in addressing DFV, and Lutheran Christian beliefs and practices pertaining to DFV. The document analysis also informed interview question schedule design.

## Literature Review

The relationship between religion and DFV warrants ongoing attention given the evidence of how religious and spiritual beliefs and practices, including uses of Christian scripture and church-based cultures, have been used to support male violence and abuse against women in intimate partner relationships (Knickmeyer et al., 2010; Marsden, 2018; Nason-Clark et al., 2017; Pepper et al., 2021). Research also demonstrates how religious practice and faith-based community membership can potentially support victim/survivors to manage and recover from the impacts of DFV, including church leaders recognizing DFV, supporting victim help-seeking and connection to other support services, safety, and recovery (Bent-Goodley & Fowler, 2006; Pepper et al., 2021; Wendt, 2008; Wendt & Zannettino, 2015).

There is some recent albeit limited research that considers the role of churches, religious leaders, and faith-based organizations in engaging with male perpetrators of intimate partner violence, supporting their accountability, and research that has involved men of faith who have abused their partners. Drawing on their extensive research with male perpetrators and a range of professional program staff, Nason-Clark et al. (2017, p. 34) discuss how clergy relate to male perpetrators, noting the significance of their hope for men to change; however, they may “overestimate” (as may Christian female partners of male perpetrators) readiness to change. Religious leaders may be challenged in their response to both victim/survivors and perpetrators as they may wish to uphold Christian ideals, including marriage (Nason-Clark et al., 2017, pp. 34, 38). They may also be at risk of being manipulated by perpetrators and therefore collude with them (Nason-Clark et al., 2017, p. 1). Male perpetrators of violence and abuse may not recognize religious leaders or their faith communities as sources of support (Levitt et al., 2008). Without training leaders may not recognize the extent of the abuse and provide inappropriate advice that does not address a victim's support and safety needs (Nason-Clark & Fisher-Townsend, 2015). Nason-Clark et al. (2017) write:

What pastors should not seek to do is to replace the role of the police or the courts. The primary roles of the pastor or religious leader are to ensure the immediate safety of the victim and to provide long-term spiritual care for victims and their family members. The pastor who seeks to confront the abuser about the violence or to enforce better behaviors within the marriage will most likely put the victim in even greater danger and will make it more difficult for the victim to find strength in her faith to escape the abuse. (p. 17)

As providers of pastoral care, the research literature has frequently reported that religious leaders are concerned they have inadequate knowledge in responding to congregation members experiencing DFV, and thus researchers have identified training needs as a critical component to supporting religious leaders to adequately provide support to victims/survivors and provide referral to relevant supports and services (see Nason-Clark & Fisher-Townsend, 2015). However, religious communities and leaders themselves may self-identify their training needs in preventing and responding to DFV, as evidenced in investment in prevention and training activities, and resource production (see Davis et al., 2021; LCA, 2017; Wendt et al., 2023b).

Secular service providers may also not recognize the significance of religion in providing supports to victims/survivors due to assumptions about the detrimental role of religion and the concern religion is contributing to the violence and hence has no role in supporting the victim/survivor (Nason-Clark et al., 2017, p. 14; see also Nason-Clark & Fisher-Townsend, 2015, pp. 150–151). Service providers may also not recognize or be respectful of male perpetrator faith needs (Nason-Clark et al., 2017, p. 78). Conversely religious leaders may hesitate to work with supports outside their faith community.

In their research Nason-Clark et al. (2017) describe abuser manipulation of religious concepts while also noting “the Christian faith also teaches that we can live differently today than we did in the past. The broad Christian narrative of conversion—repentance, remorse, altered behavior, and forgiveness—can be held as a spiritual strategy for change” (p. 84). Nason-Clark and Fisher-Townsend (2015, pp. 162–163) conducted research with participants in a faith-based batterer intervention program in Oregon. They looked at how perpetrator accountability can also be understood from a Christian perspective, can involve religious leaders and congregations, as well as a range of program interventions and supports.

Other research by Davis et al. (2020) that includes Latino, Hispanic, and Mexican male perpetrator experiences of a Catholic parish-based and voluntary partner abuse intervention program in Chicago, also noted the significance of faith in supporting an alternative and culturally relevant program design for men. Thus, there is evidence religious identity and practice and religious organizations, members, and leaders, can support both victims/survivors and perpetrators in their recovery journeys, and provide male abusers motivation to change their ideas and use of violence as well as accountability (see also Bent-Goodley et al., 2015).

Other researchers have argued that understanding religion also contributes to the social and cultural context enabling male violence against women. The issue of how religion more broadly intersects with and enables gender identities and gender relations, has also been considered. For example, Avishai et al. (2015, p. 13) argue the intersection of gender and religion includes understanding religious femininities and masculinities, and argue that given the significance of the relationship “it may be productive to think about how gender and religion constitute each other.” In their research with Christian and Muslim women in Norway, the United Kingdom, and Spain, through interviews Nyhagen and Halsaa (2016) explored women's experiences of religion, citizenship, and gender. As part of their research, the authors critique the “secular-religious binary” (p. 4):

To disentangle what stems from and/or is related to religion or to secularism, as if the two were wholly separable, is a challenging task, not least because what counts as religious or as secular is shifting in different contexts (Beckford, 2003). (Nyhagen & Halsaa, 2016, p. 34)

This point draws attention to the significance of how we understand researching “context”—in this case the religious context to DFV—and this involves allowing for the diversity of religious experiences that include a range of public and private discourses, expressions, and contestations.

Further, religious and gender identities and lived experiences are coexistent with other and intersecting aspects of identity (Nyhagen & Halsaa, 2016). The “religious context” is also spatial and includes ecclesiastical and religious organizational and community expression of gender, sexuality, intimacy, gender relations, and gender equality. It is also interactive with, nested within and informs other gender orders (local, national, global) (see Connell, 2005), as well as state-based conceptualizations of—and responsibilities for supporting—gender equality (see Nyhagen & Halsaa, 2016) and addressing DFV and supporting safety (Franzway et al., 2019). This article contributes to the importance of understanding such gendered identities and lived experiences within religious contexts, to explore how they shape DFV and responses to it.

## Methodological and Theoretical Considerations

A strategy and framework were developed to inform the first stage of the research project: document analysis of key LCA documents in the public domain. The purpose of the document analysis method is to understand the theological drivers of the LCA's understanding of DFV and its efforts to address DFV including policy and practice reform. The document analysis work and findings informed subsequent data collection strategies for the larger project.

Feminist poststructuralist theory supported the document analysis work. That is, the document analysis employed in this research project was used as a feminist research practice seeking change. For this project, the document analysis not only sought to contribute to a body of knowledge about religion and DV by identifying and focusing on theological drivers, but also sought to support transforming the LCA's practice in preventing and responding to DV. The body of knowledge produced, will enable the larger project to provide recommendations to the LCA to improve its response practices including to male perpetrators. Thus the researchers and the LCA as funding partners are seeking transformation together. Poststructuralist theory can inform and guide document analysis work. It enables analysis of issues, experiences, institutions, and discourses, with a focus on understanding power as relational, and further, supports insights into how power and knowledge are connected. Poststructuralist theory can also identity constructions of “truths” that have impacts on everyday lives, informing embodied practices, social and gender relations, and regulation and governance including that practiced by the LCA (Foucault, 1980; Hall, 2001; McLaren, 2002).

Specifically, this research engaged with Foucault's approach to theorizing and analyzing discourse as the interest was to gather insights into LCA documents and theological decrees that express the conceptualization of power and knowledge guiding Lutheran practices with respect to gender relations, gendered subjectivities and importantly, the “problematization” (Bacchi, 2009) of DV in a Lutheran religious context. As Hall (2001) describes:

Discourse is about the production of knowledge through language … It is about language *and* practice. It attempts to overcome the traditional distinction between what one *says* (language) and what one *does* (practice). Discourse, Foucault argues, constructs the topic. (p. 72)

It is also important to note that Foucault's writings show how discourses have a historical context, and may be challenged and so change (McLaren, 2002, p. 31).

## Method and Analysis

Bacchi's (2009) “What's the problem represented to be?” approach to policy analysis was useful to support the document analysis method. Bacchi's (2009, p. xii) method includes six questions to problematize policy or rules and aims “to interrogate the **problem representations** that lodge within public policies in order to see what they include and what they leave out” [original emphasis]. While the full extent of documents analyzed for this research also included theological and church-member articles and letters, Bacchi's approach to policy analysis remains useful to inform a critical engagement with policy and theological documents produced by the LCA.

Similarly, Bryman (2016, p. 561) argues “documents need to be recognized for what they are namely, texts written with distinctive purposes in mind, and not as simply reflecting reality.” Researchers need to consider the narrative and meaning of a document as well as be clear about how and why they are using documents, matters of intertextuality and how documents relate to each other and further, researchers need to be reflexive (Coffey, 2013).

Several questions were therefore developed to guide the document analysis:

- How are the documents describing and representing DFV?
- How do narratives about and representations of DFV change over time?
- How are the documents representing family life, intimacy, and gender relations?
- How do the documents represent gendered subjects—including but not limited to femininity and masculinity?
- How are the documents representing who is impacted by DFV, for example, women, women and men, children, congregation?
- Are differences of opinion within the LCA described and/or critiqued?
- How is safety, duty of care, and responsibility articulated?
- How are supports, services and help-seeking strategies being discussed?
- Is gender equality discussed?
- How does the LCA articulate its role and strategy in addressing the problem of DFV?
- In all of these points, what specific Christian Lutheran beliefs, scripture references, and practices are being referred to, directed at whom and for what use and outcome?

Choices are made in the process of document analysis work, and we agree with Bacchi (2009, p. 20) who notes that text selection is an “interpretive exercise.” Understanding the relationship between religion and DFV required reflexivity in the research practice including attention to how religion may influence practices of violence and abuse, as well as how it informs supports for victim/survivors and their recovery, and also, opportunities to provide support for perpetrators and their recovery and accountability. The scope of research exemplifies both concern for the misuse of religion to contextualize and engender DFV as well as, the capacity for religion and faith to offer hope, safety, and justice in responding to DFV and helping to end, violence against women. Documents analyzed included detailed discussions of theology and hermeneutics regarding gender roles and research team members drew on their various disciplinary backgrounds of social work, sociology, and theology as well as their religious backgrounds, to confer on clarifying and interpreting critical theological concepts and arguments, and how to manage findings. A key issue identified was navigating feminist research practice respectful of the range of lived experiences of Lutheran religiosity as expressed through documents, and how to report on findings that make important associations regarding the relationship between gender equality and the Lutheran religious context to DFV. As researchers we assume gender equality is required to support ending DFV and violence against women (see Our Watch, 2021). What helped the reflexive process was returning to the guiding intent of the research: the LCA invested in the research to support improving its capacity to understand and address DFV.

### Sample

Alongside the importance of acknowledging interpretive and reflexive work, the framework developed supports consistency in research practice analyzing documents. In total the document analysis sourced documents from the LCA's website, the *Lutheran Theological Journal*, The Lutheran (an LCA publication), and considered documents suggested by the research project's partner Chief Investigator representing the LCA^6^ and those documents suggested and provided by the LCA's DV Taskforce membership. In this article, we overwhelmingly discuss findings from analysis of publicly available LCA documents.

**Table:** 

Documents produced by the Lutheran Church of Australia	
Type of document	Number
Doctrinal Statements and Theological Opinions	6
LCA Domestic Violence Campaign documents (includes website)	3
Internal documents prepared by the LCA DV Taskforce	4
Other LCA documents includes documents from General Synod meetings; a document from the Commission on Social and Bioethical Questions; and a discussion paper regarding same sex relationships within Lutheran aged care.	4
LCA policy documents	5

## Findings

Building on the method used to support analysis of documents produced by the LCA, in this article, we present findings around four key themes. First, we demonstrate how the LCA is naming the problem of DFV and the history of this effort. Second, we consider representations of gender relations and DFV and explore how safety is represented and discussed within the LCA. Third, we consider questions of gender equality that are raised through an examination of relevant LCA documents. Finally, we discuss how key LCA documents are constructing and positioning the gender identity “woman.”

### Naming the Problem of DFV

Document analysis showed that the LCA has a history of naming the problem of DFV, raising concerns about the issue and its impact, and developing initiatives to identify and respond to it. Over time, the trajectory of official LCA engagement with identifying and responding to DFV has transitioned from a broad-based statement acknowledging the significance of the issue, to the current LCA DV campaign involving multiple strategies to prevent and respond to DFV, support safety and manage risk.

In 1993, the LCA's Commission on Social and Bioethical Questions prepared a one-page statement on DV that was adopted by the General Synod later that year (LCA, 1993). This document is a resolution by the LCA acknowledging the prevalence of DV in the Australian and Lutheran community. The resolution acknowledges that DV “has been defended as Christian discipline and the legitimate exercising of Christian authority” and goes on to condemn “all forms of violence in the family.” Synod delegates resolved to support and respond to both victims and perpetrators of violence/abusers. This is a short statement condemning violence and “call[ing] on people to live in the peace of Christ in their relationships in the family.” At that time with the 1993 statement, the LCA makes a clear acknowledgement of the problem of DV, that this has occurred and been defended in a Christian context, that the LCA has a role in addressing the problem and supporting both victims and perpetrators, and resolves for education that demonstrates the Bible supports the equality of men and women.

At the LCA's General Synod in 2003, a resolution was made following the agenda item titled “Creating a culture of peace.” The resolve details actions for the (then) current decade and thereafter, including supporting the availability of resources and “speaking out against” family violence; “material, emotional and spiritual support” to those experiencing violence; and promoting seminars about DV with an awareness and prevention focus (LCA Inc., 2003, p. 26).

The LCA is currently implementing a campaign to prevent DFV, titled “Hidden Hurts Healing Hearts.” The campaign was established following a resolution made by the General Synod in 2015:

That the Convention of Synod reaffirms its condemnation of all forms of violence in the family and authorises GCC [General Church Council] to commit resources for a church-wide campaign to address the prevalence of Family Violence amongst us, which may include sharing of resources, education initiatives and the provision of pastoral care to the survivors of violence, as well as the perpetrators of abuse. (LCA, n.d.)

The General Synod also resolved for the LCA's Commission on Theology and Inter-Church Relations (CTICR) to:

study the Lutheran theological and scriptural understanding of subordination and the role of male headship in marriage and the contextual implications for family violence. (LCA, 2018, p. 255)

The LCA established a Taskforce on DV in 2017. Similar to the 1993 resolution, in current LCA campaign documents it is recognized there is a need to support both victims of violence and perpetrators of violence, and to support families. Campaign documents have clear messaging about prioritizing the safety of women and children. They also define and describe in detail types of DFV, and discuss the impacts. Victim/survivor safety, risk, and perpetrator accountability are all key considerations in campaign documents.

The 2017 *Domestic Violence Handbook for Pastoral Workers* offers examples of questions to support pastor and pastoral worker responses to victims/survivors and perpetrators, and promotes understanding limits to the support that can be provided and the need to work with service providers. The Handbook describes how responding to DFV within the church includes knowledge of, and relationships with, services outside the church. It is important to note the reference to pastoral support and care as intrinsic to efforts to recognize and respond to DV, and the Handbook offers guidance on safety planning with victims to support and prioritize their and family members’ safety. The campaign booklet discusses how victims of DV need to be supported to feel safe in their faith community. The Handbook also clarifies that perpetrators make a “choice” to use violence, and makes suggestions as to what constitutes taking responsibility for violent behavior, and how to engage with and respond to perpetrators and support victim safety. The latter includes consideration of how a perpetrator may remain in a faith community or be referred elsewhere. Thus, supporting victim and family safety is understood as a responsibility across the church in that safety is supported by individual pastoral care and response, as well as by increasing congregation and faith community capacity to understand and respond to DV.

### Representing Gender Relations and DFV

The LCA's DV campaign's website offers more practical information on seeking support and getting help, and useful contact information, information about lived experiences, and links to resources. Included in the LCA campaign narrative is an emphasis on a commitment to transformation, to reducing the occurrence and impacts of DFV and the need for change within the church. More specifically, it is worth noting that the campaign acknowledges that there have been inadequate past practices by the church and interpretations of scripture supporting male power, violence, and abuse. For example, the LCA's DV prevention campaign website (LCA, 2020) describes how:

Too often past practices in church communities were built on false myths of male domination and control. And too often this meant that men were excused for their violent and abusive behaviour, and women were blamed and/or forced to return to very unsafe relationships. It is therefore imperative that we re-examine the ways in which we provide support to victims of abuse, and challenge the values and behaviours of those who perpetrate it.

Furthermore, the campaign documents (including the website) also describe the need for a theological underpinning to change and challenge male behaviors, and recognize the misuse of New Testament scripture, in particular Ephesians 5:21–25^7^ which references male headship and marriage, and submission in marriage. The campaign website, for example, describes how scriptural references to submission and subordination (and male headship) cannot be used to support abuse:

Any man among us who uses Christianity and the Bible to justify abuse of his wife or partner has clearly lost sight of his faith. If we are to use the word ‘subordination’ at all, it must relate to Christ's voluntary submission to the will of his heavenly Father when he went to the cross. Such submission is freely given and never demanded. It's a loving expression that marks the difference between Christians and the world (see the contrast Jesus establishes in Matthew 20:25–27). That does not mean that we encourage people, particularly women who at risk [sic], to stay in abusive relationships. We plead with such women: please actively seek help and support to protect yourselves and your children. (LCA, 2020)

This quote from the campaign's website indicates the concern about understandings and uses of Ephesians 5:21–25, and how selected scripture has been used to support inaction on DV, as well as support a man's/husband's power, control, and violence over and against a woman/wife.

The Domestic Violence Handbook for Pastoral Workers also discusses the (mis)use of Ephesians 5:21–25. Again, it is argued that (male) headship does not equate with “dominance.” Further, neither male headship nor dominance are referred to in the LCA Statement on Marriage, Divorce and Remarriage—a point also made in the campaign's website and brochure. The Handbook discusses how practicing forgiveness does not mean one needs to accept and live with violence; which is largely a role women have been expected to fulfill. The campaign's brochure also refers to the concept of forgiveness and additionally, refers to repentance—the latter “is much more than saying sorry. True repentance will be shown in changes in attitude and behaviour” (p. 6).

In considering the LCA's campaign documents and statements on DV and its impacts—including definitions of spiritual abuse—it is clear that prevention and response strategies prioritize victim safety and support addressing injustices through a faith-based lens. Scripture of concern is cited, particularly Ephesians 5:21–25 and the concern is the misuse of scripture by perpetrators and the wider church community whereby abuse has been justified and condoned. Campaign documents deliver the clear message that Ephesians 5 21–25 is not to be interpreted as justifying male power over women or violence against women. Other scripture cited in campaign documents offers hope and support to address DV as a matter of faith, and provide evidence that Jesus treated women equally.

Concerns with the misuse of Ephesians 5:21–33 are also expressed in the CTICR Taskforce on Domestic Violence 2018 report prepared for the General Convention of Synod. As previously cited, at the General Synod in 2015 the church passed a resolution that the CTICR will study understandings of subordination and male headship with respect to “the contextual implications for family violence.”

The Taskforce 2018 report and campaign documents identify and address the risk of the use of select scripture to condone and justify violence against women. Indeed, in the report the authors ask the question “Have Lutheran Theological Understandings of subordination and male headship contributed to male violence against women in the church?” This is the risk identified and it is a theological matter of concern to the LCA as expressed through the Synod statements and campaign documents. The authors of the Taskforce report conclude “no public theological statement or position of the LCA consciously condones or justifies violence against women” (LCA, 2018, p. 256) yet also note scripture has at times been used to justify control and abuse of women. The Taskforce report also goes on to discuss repentance and forgiveness and recommends further study “in the context of violence in relationships” (p. 257). Finally, in providing the clear statement that violence against women and in marriage is unacceptable, the report poses several questions that further problematize the relationship between Lutheran theology and practice as the church seeks to address the problem of DV:

Is more needed, however? Would the LCA benefit from a clear theological and pastoral statement renouncing violence in the home and being clear about the servant nature of male headship in marriage?

In what ways might our churches systemically, though unintentionally, cooperate with abusive persons, helping them to justify and perpetuate their abuse?

Is it possible that an all-male clergy may in some way contribute to the conditions that allow domestic violence to continue? For instance, through lack of experience or awareness, could male clergy fail to take domestic violence seriously, or be manipulated or trapped into colluding with male perpetrators? Is it more difficult for women to disclose domestic violence to their pastor because he is male? (LCA, 2018, p. 258)

The Taskforce report demonstrates the significance of the key religious concepts of forgiveness, repentance, male headship, and subordination as it engages in a theologically inspired reflective process to understand the Lutheran context to the perpetration of intimate partner violence.

### Questions of Gender Equality

The church has clearly identified it has a role in offering pastoral support to victims/survivors and perpetrators of violence, and supporting safety and change. Yet the LCA is diverse and theological understandings of gender relations—and uses of scripture—are also diverse. General Synod resolutions and LCA DV campaign efforts are nested in other LCA theological explanations and practices regarding gender relations, roles, and responsibilities which we now discuss. These are often at variance with the desires that the LCA DV campaign expresses. Together these documents and the LCA DV campaign are revealing when read intertextually.

The LCA does not permit women to hold the office of ministry. The rationale for this practice is detailed in the publicly available Doctrinal Statements and Theological Opinions^8^ (DSTO) document *Theses of Agreement VI: Theses on the office of the ministry*. This document was established in 1950 and reviewed in 2001 “unedited.” It describes how the New Testament defines matters of ordination and how the ministry is “an office instituted not by man, but by God.” Specific New Testament scripture is cited to justify the LCA's position that women cannot be ordained:

Though women prophets were used by the Spirit of God in the Old as well as in the New Testament, 1 Cor 14:34,35 and 1 Tim 2:11–14 prohibit a woman from being called into the office of the public ministry for the proclamation of the Word and the administration of the Sacraments. (LCA, 2001a)

This document together with a group of DSTO documents on women in the church, establish an LCA discourse where *women are a problem* in that documents discuss women's exclusion from public ministry thereby referencing the matter of women and ministry is problematic and requires a focused explanation and justification, and establish the opportunities they do have available to them, for example, as Sunday school teachers (LCA, 1978/2001a, 2001b). The problem *is* the matter of gender roles and opportunities within the church and specifically opportunities for women, and the explanation is that exclusion and inclusion opportunities for women are determined by selected New Testament scripture.

*Thesis VI.11 on the office of the ministry* refers to New Testament scripture to justify the exclusion of women from public ministry. Hermeneutics and the inerrant standpoint of an unproblematic and fixed interpretation of selected scripture establish a Lutheran patriarchal organizing structure and authority over women. Responsibility for the *process* of determining this official LCA theological position that involves *interpreting* scripture and *practice* resulting in a gendered church structure defining Lutheran gender relations and roles is never allocated to individuals rather, responsibility is deflected to the Word of God. To be clear: this is official Lutheran theological discourse *and* institutional practice of social exclusion providing instruction to the reader and church membership. It is about women and men as cumulatively, DSTO documents establish a heteronormative and patriarchal church structure informing gender relations, determining and restricting opportunities for women. Official DSTO documents establish women categorically and, also by implication, a Lutheran male identity is established as entitled—to the opportunity of public ministry, authority, and in relation to women. A Lutheran patriarchy is normalized—ontologically and epistemologically—as official discourse secures and restricts the meaning and use of selected scripture as well as gender categories and roles (see Butler, 1990).^9^ Women are othered in relation to men and discrimination on the basis of sex/gender is practiced. The social norm of patriarchy as expressed in DSTO discourse and deferral to select scripture in referencing women, values a type of Lutheran male ministry and masculinity that discriminates against women. In this Lutheran-as-religious-institution discursive space it is hard to name and challenge the impacts and effects of discrimination if responsibility for social and gender norms is hermeneutically organized and justified as fixed meaning in reference to the New Testament. The male/female binary opposition is theologically secured using scripture to determine not only public ministry but gender roles within the Lutheran church. The issue of gender equality regarding public ministry is inferentially named in the justification for male-only public ministry and then silenced in the official LCA theological argument that separates a theological determination within the organization of the church from any reference to the lived experiences and impacts of discrimination on the basis of gender. Representation of scripture is separated from the use, experiences, and impacts of scripture as well as from any contested interpretations of meaning and use.

Again *the problem of women* is named in the LCA's Theses on public ministry when it details the theological rationale for excluding women. Moreover, the DSTO document *Volume 1 F. Women in the Church: The role of women in the church* (LCA, 1978/2001a) further articulates how women are positioned in relation to men and in the church as the organizing structure informing faith communities and influencing gender relations, marriage, and family life. This DSTO document again argues the matter of the role of women in the church is determined by a selection of scripture: “The sacred Scriptures of the Old and New Testaments are the only source of teaching and right practice in the church” (LCA, 1978/2001a, F3). The DSTO lists examples of women who served Jesus Christ and then stipulates “However, none of these women worked independently. They were helpers, supporters of the Lord and the apostles.” The document goes on: “It seems that when the disciples were alive there was no real problem about the role of women in the church” (F3). This claim there was no real problem is revealing of the current problem for the LCA—the problem *is* women and gender inequality in the church, inequity of opportunity, and the matter of male-only public ministry.

This DSTO document on the role of women goes on to detail New Testament scripture that supports the LCA's policy and practice regarding opportunities for women in the church:

The passages which come into consideration are 1 Corinthians 11:2–10; 14: 33b–36; and 1 Timothy 2:13,14. These lay down the principle of subordination and reserve for women in the church. Or to put it negatively: women are not to take a leading, independent, authoritative role in the church. Note: a congregation of women would, of course, have to have women officers. (F4)

Women are being positioned as helpers and supporters in relation to the Lord, the Church, and men. The LCA uses the theological concept of subordination as per the cited scripture to support this position for women, for the practice of denying women equal opportunity in the church. This is an expression of power over women by the church—the “church” includes those individuals that have determined the DSTO's and overarching and guiding Lutheran theological decrees—that cannot be disassociated from the practice and lived experience of gender relations whereby the LCA is asserting men have power over women. The “church” here is not empty of individuals, embodied processes, and power relations. Women are being denied equal leadership opportunities within the church and also, are being positioned as subordinate to men. The theological argument for the principle of subordination—for this document is an argument notwithstanding the discourse that represents scriptural interpretation as fixed meaning—has a consequence that connects church-based practice with gender relations within the church.

### Constructions of Woman

The DSTO document *Women in the Church: The role of women in the church* is one of three DSTO documents on the role of women in the church that together contribute to a broader LCA discourse that is patriarchal in the theological advice and instruction provided to the reader. The DSTO document *WOMEN IN THE CHURCH Statement on rights of women to vote at meetings of the congregations* (LCA, 1966) was prepared in 1966 and has not since been edited. The document commences with reference to scripture that establishes a sex/gender binary difference:

In Christ man and woman have equal standing, Mark 12:25 and parallels, Galatians 3:28; but there is a difference between man and woman by virtue of the fact of creation, 1 Corinthians 11:7–10; 1 Timothy 2:13; Genesis 2:18ff, by which a subordinate position has been given to women. A further reason is the role played by the woman at the Fall, Genesis 3:1ff; 1 Timothy 2:14. This subordination shows itself, as far as the individual woman is concerned, in the marriage relation, Genesis 3:16. (F1)

With respect to relations within the congregation, the document considers scripture as it pertains to the role of women:

4. Since the Apostle has in mind the worshipping congregation, the divine service, as the place for which his order is meant, it is improper by way of deduction to claim silence of women in all congregational, business, and social meetings.

5. By giving woman as a member of the congregation the right to vote she is not necessarily given authority over the man, as has sometimes been claimed. (F1)

The document goes on to detail that “congregations may grant women the right to vote at congregational meetings” (F2). A resolution from the General Synod in 1968 clarifies the point that subjection can be “safeguarded” as men and a male vote may make a final decision on a matter. Here the “congregation” is defined as having a collective male identity as well as men having discretionary decision-making power over women within the congregation.

The LCA DSTO document Volume 1 H. *ETHICAL AND SOCIAL ISSUES Marriage, divorce and re-marriage* (LCA, 1978/2001c) defines marriage as “the union of a man and woman,” defines the purpose of marriage and addresses divorce and remarriage—all points are evidenced with scriptural references. In this document marriage is defined as heterosexual and an ideal is presented, one where divorce is to be avoided and pastoral counseling involves efforts to support a reconciliation so a marriage may continue, although divorce is permissible in certain situations. Notions of forgiveness, repentance, and love are key concepts guiding a Lutheran understanding of marriage.

Marriage is again discussed in the DSTO document Volume 1 H. *ETHICAL AND SOCIAL ISSUES Human sexuality: three key issues* (LCA, 2015). This summary document discusses human sexuality and marriage and asserts same-sex relationships do not demonstrate marriage. The document also discusses the breakdown of marriage specifically referencing DV:

God's intention for marriage is that it be lifelong. … God's will is most fully lived out when couples who experience conflict live in repentance and forgiveness. At the same time, the LCA can never condone abusive relationships, and places a high priority on protecting victims of abuse from harm, when conflict between a husband and wife becomes excessive. (p. 3)

This reference to abusive relationships is noted among the DSTO theological discussion asserting—and limiting—Lutheran sexuality, gender and marriage identities and relations. A wider reading of DSTO documents indicates challenges to Lutheran social and gender norms and LCA concern about the issue of DV within marriage, in the context of theological determinations of what are permissible Lutheran intimate partner relations.

Yet the theological rationale driving the structure and organization of the LCA with respect to male-only public ministry is contested at the same time it is maintained. While DSTO documents are publicly available on the LCA website, the website also contains information about the history of efforts to ordain women in the LCA, efforts that are more recent than the date of the above cited DSTO documents. Thus, the authority of these documents shifts considering intertextuality and how they connect to documentation of LCA efforts to seek transformation of the ministry and gain the support of the Synod to ordain women. The Synod has voted five times in total on the matter of ordaining women—in 2000, 2006, 2015, 2018, and 2023. The most recent vote to ordinate women (unsuccessful) was then followed by a proposal for the LCANZ to undertake a process to consider two practices of ordination within the church (proposal accepted). Further, comparative analysis of LCA documents—DSTO and DV campaign documents, for example—demonstrates efforts within the church to support gender equality and concern about how a range of selected scripture has been used supporting DFV, and various types of violence including spiritual abuse, and the impacts including its consequences with respect to normalizing and condoning violence against women.

## Discussion

The power and authority of men over women is established and reaffirmed through a collection of official documents stipulating theological organization of the church, marriage, and ministry. However, LCA discourse on gender relations and the role of women and men is not singular—there are diverse views and a disruption and a process of engendering change, for example, expressed through theological arguments, DV campaign documents and Synod debate and votes on the ordination of women. Further, the DV campaign is supported by the LCA Synod.

The analysis of documents discussed in this article also reveals disagreement within the LCA regarding differing interpretations and use—and impacts—of the theological notions of submission and male headship, in particular. This in turn requires reflection upon to what extent official and organizing theological decrees effect the distribution of power in gender relations as the document analysis presented in this article also illustrates a culture of care within the LCA in addressing and ending DV. It is a limitation of this article that it does not include findings from analysis of other types of documents including other Lutheran publications that further exemplify the nuances of change occurring within the LCA, efforts to support safety, inclusion, and gender equality, as well as lived experiences of Lutheran gender relations.

However, given the DSTO documents on the role of women are still current—as is the Theses of Agreement analyzed above, an LCA dominant discourse prevails—albeit challenged—where women are positioned as secondary in a gender/sex binary that privileges a type of Lutheran male identity entitled to public and private leadership, voice, space, more power than women with respect to voting and participation, and so consequently, power over women. After all, documents focused on “women” are consequently, focused on men and gender relations. Further, that there are documents under the heading the “role of women” positions a LCA subdiscourse about the role of women as distinct from the main business of the rest of the LCA DSTO matters expressed through the DSTO documents, which is the business of men. This framing of women and their role in the LCA—as expressed in DSTO documents—establishes limits on opportunities for women and a singular expression of femininity in relation to men within the church, congregations, and families.

Current LCA theological priorities support a patriarchal church structure and support gender relations and lived experiences within. While the relationship is authoritative it is negotiated and challenged. As the LCA DV campaign notes, there are some current practices and use of select scripture that are of concern because they indicate Lutheran practices that may support violence against women and gender inequality. The LCA's DSTO documents cited above do not consider the effects and impacts of the theological statements. There are at least four matters of concern here: first, that by virtue of their gender women are represented as a problem and divested of power and equal opportunity *within the church, congregation and in relation to men*. Second, that in the argument for discriminatory practices the LCA devolves itself of responsibility for the impacts of the gender inequality it supports as it does not consider the lived experiences of DSTO positions, instead arguing for the infallibility of the use of selected scripture. Third, DSTO documents defining the role of women establish opportunities, leadership, and power for men, through theological arguments that avoid scrutiny of the process that engenders a Lutheran masculinity entitled to opportunities denied to women. Fourth, the LCA's position on the role of women in the church through the use of selected scripture, establishes a religious, social and cultural context that may place women at risk of violence. Prioritizing use of scripture that positions women as subordinate to men in the church structure, organization, and congregation, risks supporting male entitlement to power and control over women in intimate relationships (see Flood, 2011; Ogden, 2021; Our Watch, 2021).

Elsewhere research into violence against women has highlighted the detrimental impacts of women's harassment in and exclusion from public spaces. Vera-Gray and Kelly (2020) in their research on the sexual harassment of women in public spaces and the “safety work” women and girls do, make the point that gender relations are contested in public spaces. We draw attention to this matter of space, as within the Lutheran church the office of public ministry can be viewed as a public space being contested through efforts within the Lutheran community in Australia to support the ordination of women and this is a matter of supporting gender equality and social inclusion within the LCA. While ministry remains a male-only domain, the extent to which this Lutheran practice and theological determination supports gender inequality within the church but also in families, is a concern. After all, theological claims for practicing exclusion on the basis of gender are not inseparable from practices where men have theological reasons for claiming power and control over women in intimate partner relations and private spaces. To make connections between spheres of official theological determinations, religious and church activity, marriage and family life, is to seek to understand the Lutheran religious context informing risks, as well as opportunities for the LCA to further support addressing violence against women.

The LCA has identified the problem of DV and seeks change through establishing a campaign to contribute to safe relationships. The church has adapted and expanded its understanding of its responsibilities in identifying, responding to, and preventing DV. Official theological determinations informing the organization of the church cannot be disaggregated from efforts needed to support gender equality and reduce violence against women in religious communities. For the LCA this involves acknowledging the theological arguments informing Lutheran masculinity as well as femininity, and the impacts and experiences of those theological arguments for Lutheran gender identities and relations.

Current use and interpretations of the theological concepts of headship, subordination and order in creation in official LCA policy^10^ and practice are informing the social and organizational context to the current LCA campaign to address family and DV. There is the risk of confused messaging about safe, peaceful, and respectful gender relations including—but not limited to—the risk posed to women (and children) if women continue to be denied equal opportunities *within* the church as a religious organization but also, the church as an entity is providing context and support to congregational and family life.

## Conclusion

This article analyzed documents produced by the LCA, to understand the church's initiatives to recognize, prevent, and respond to DFV. We have also considered key LCA documents that articulate the church's understanding of gender roles and responsibilities. From these documents, we have extrapolated key ideas about Lutheran gender relations in the Australian context, and how they are informed by scriptural references and uses. Our findings demonstrate the LCA's current ecclesiastical framing of gender relations support a religious context limiting gender equality at the same time the church endorses and implements a campaign to address DFV. While the church is reflective as it increases its capacity to challenge DFV within its religious community, and offer support to both victim/survivors and perpetrators of violence, the intersection of its DFV campaign with religious organizing practice that limits opportunities for women, and theological determinations and arguments for truths based on scriptural interpretation that restrain gender equality, needs further recognition to increase support for safe and peaceful intimate partner relations.

Theological drivers of church-based gender and social norms that discriminate against Lutheran women and *limit equality* in gender relations through discourses and practices “othering” women, are at risk of normalizing and condoning unequal relations between women and men in intimate relationships. There is the opportunity for the LCA to revise its theological priorities and practices *throughout the church*, to support the campaign's intent but also, extend the LCA's capacity to promote safety and equality (for—but not limited to—women and men; in relationships, families, congregations, workplaces) and address current injustices on the basis of gender. Finally, this opportunity to understand the connection between efforts to end DFV and the religious context, and how this is linked to supporting gender equality, is one that extends to other denominations and faiths; it contributes to understanding how religious organizations and communities have a critical role in ending DFV and violence against women. The findings illustrate that when we talk about the relationship between religion and DFV, we need to consider diverse religious institutional arrangements and how they have and exert power, including how religious institutional structure and relations within, may influence gender and intimate partner relations, and shape attitudes towards DFV including support for recognition, prevention, and recovery.

## Notes

1. In this article, the term “family violence” is referring to intimate partner violence. While family violence is a term that also refers to violence committed by a family member to another, here both the terms “domestic and family violence” are used to acknowledge cultural differences in preferred language use to describe intimate partner violence.

2. The terms “religious” and “faith-based” are used interchangeably to recognize the diversity of religious and spiritual practices, identities, institutions, and denominations, and strive for language that is inclusive.

3. It is important to note that religious and faith-based organizations have also developed policies and practices responding to child sexual abuse. In Australia, the Commonwealth Government conducted a 5-year Royal Commission into Institutional Responses to Child Sexual Abuse presenting the final report in 2017. Findings include details and the impacts of the sexual abuse of children in religious institutions and leads to recommendations supporting “child safe religious institutions” (Commonwealth of Australia, 2017, p. 79).

4. The Lutheran church is a Protestant church which developed from the Reformation during the 1500s and is based on the confessional writings of Martin Luther (see Worthing, 2017). Zweck (2009) has described how “[t]he LCA is a confessional and evangelical Lutheran Church” (p. 392). As Worthing describes:
Broadly, the LCANZ is a “confessional” church because it takes as its theological foundational documents the various confessions of faith arising out of the 16th century Lutheran Reformation … The LCANZ, like other Lutheran bodies, tends not to use “evangelical” in the sense in which it has been used in the English-speaking world since the mid-twentieth century to mean “conservative”. The term evangelical … was the term that Luther and his colleagues used to differentiate their tradition from the Roman Catholic tradition. … in the LCANZ the term “evangelical” simply means “Lutheran” or “gospel oriented.” (M. Worthing, personal communication, October 17, 2023)

5. The research was conducted by researchers from Flinders University and the University of Divinity and funded by the Australian Research Council and the Lutheran Church of Australia ARC grant LP190100269. The authors thank the LCA's Domestic Violence Taskforce members who provided key documents. This article includes findings available in the research final report (Wendt et al., 2023a).

6. Professor Wendy Mayer, Dean of Research Strategy, University of Divinity. We also acknowledge and thank Dr Anna Nuernberger, Director of Research, Australian Lutheran College, for her assistance in suggesting articles from the Lutheran Theological Journal that may be relevant for inclusion.

7. Ephesians 5^21^ submit to one another out of reverence for Christ.^22^ Wives, submit yourselves to your own husbands as you do to the Lord.^23^ For the husband, the head of the wife as Christ is the head of the church, his body, of which he is the Savior.^24^ Now as the church submits to Christ, so also wives should submit to their husbands in everything.^25^ Husbands, love your wives, just as Christ loved the church and gave himself up for her (The Holy Bible, 1973/2011).

8. Doctrinal Statements and Theological Opinions are official theological statements of the LCA:
These statements are either made by the church in Convention, or produced by the Commission on Theology and Inter-Church Relations or the Commission on Social and Bioethical Questions in response to questions raised in and by the church. (LCA, 2001b)

9. Butler (1990, p. viii) writes “I asked, what configuration of power constructs the subject and the Other, that binary relation between “men” and “women,” and the internal stability of those terms? What restriction is at work? Are those terms untroubling only to the extent that they conform to a heterosexual matrix for conceptualizing gender and desire? What happens to the subject and to the stability of gender categories when the epistemic regime of presumptive heterosexuality is unmasked as that which produces and reifies these ostensible categories of ontology?”

10. For example, see DSTO Volume 1 F. Women in the church: The role of women in the church.
